# Supplementary material for: The Tryptophan-Kynurenine Metabolic System Is Suppressed in Cuprizone-Induced Model of Demyelination Simulating Progressive Multiple Sclerosis
Source: Biomedicines. 2023 Mar 20;11(3):945. doi: 10.3390/biomedicines11030945 (PMC10046567; doi:10.3390/biomedicines11030945)
Supplement: Supplementary file 1 [file biomedicines-11-00945-s001.zip › biomedicines-2196811-supplementary.pdf]

biomedicines

MDPI

Supplementary Materials

3. Results

3.1. *Investigation of Body Weight*

On the third day of CPZ toxin treatment a significant decrease was observed in the body weight of the treated animals, compared to CO, while the decreasing tendency in the body weight disappeared upon the beginning of the remyelination phase. By the end of the investigation, both CO and treated group showed no differences regarding to the body weight of the animals (Figure S1).

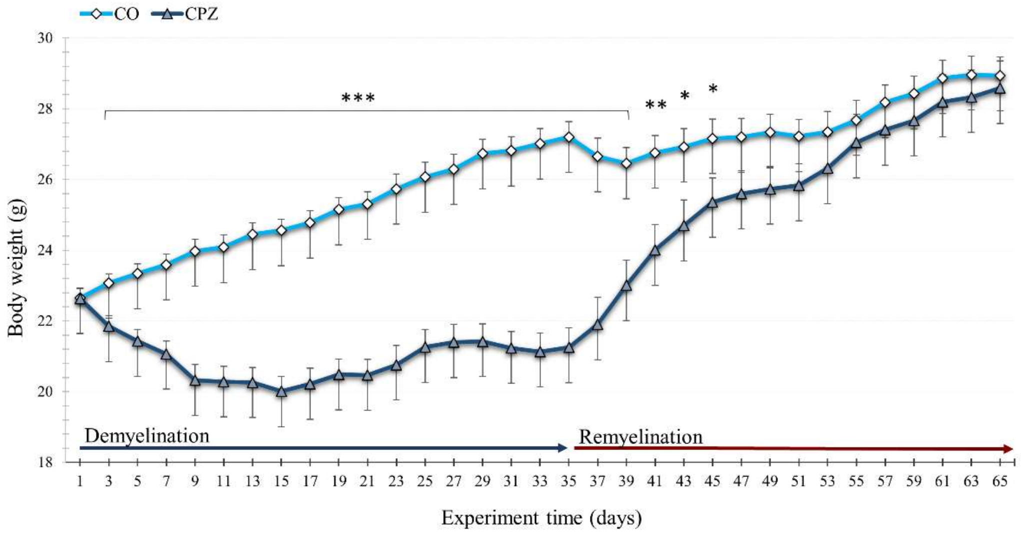

Figure S1. Alteration in body weight of the animals during the experiment. The control group is depicted with white diamonds and cuprizone treated group is depicted with white triangles. The two major parts of the experiment, i.e., the demyelination- and remyelination period are indicated with blue and red arrows, respectively. CO: control group; CPZ: cuprizone treated group, *: $p < 0.05$ vs. CO, **: $p < 0.01$ vs. CO, ***: $p < 0.001$ vs. CO. The data are presented as mean ± SEM.

3.2. *Evaluation of Cuprizone Damage in the Demyelination and Remyelination Phases*

The extent of myelin damage was examined by luxol fast blue - crystal violet staining.

As a result of CPZ-induced intoxication, the corpus callosum showed a significant demyelination as early as week 3 of treatment (acute demyelination phase), which damage was even more extensive in the 5th week of treatment. With two weeks in the recovery phase, brain samples did not show any signs of myelin damage, and nor did the samples taken in the 9th week of experiment (Figures S2 and S3).

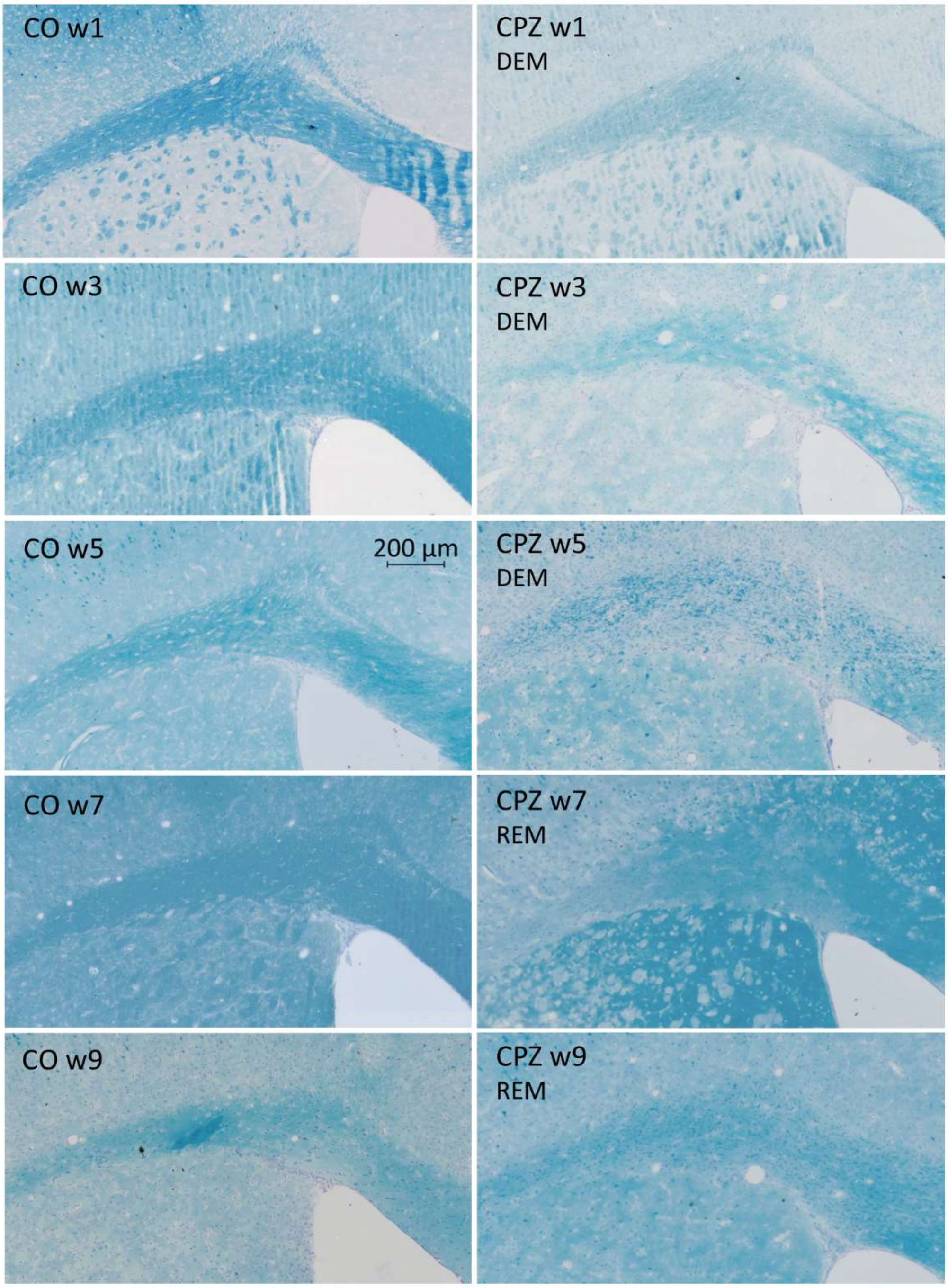

Figure S2. Luxol fast blue - crystal violet staining in the corpus callosum of the control and cuprizone-treated groups in the first, third, and fifth week of CPZ treatment (DEM), and in the seventh and ninth weeks of the experiment, which is the second and fourth weeks of the recovery phase (remyelination). A significant decrease in myelin content was observed in the third week of CPZ-treated animals (CPZ w3 vs. CO w3), compared to the CO group, which became even more pronounced by week fifth of intoxication (CPZ w5 vs. CO w5). No significant differences were observed after the animals stopped receiving the CPZ (remyelination (REM) phase, CPZ w7 vs. CO w7; CPZ w9 vs. CO w9). Scale bar: 200 µm. CO: control group; CPZ: cuprizone treated group; DEM: demyelination phase in the treated group; LFB: luxol fast blue; REM: remyelination phase in the treated group; w: week.

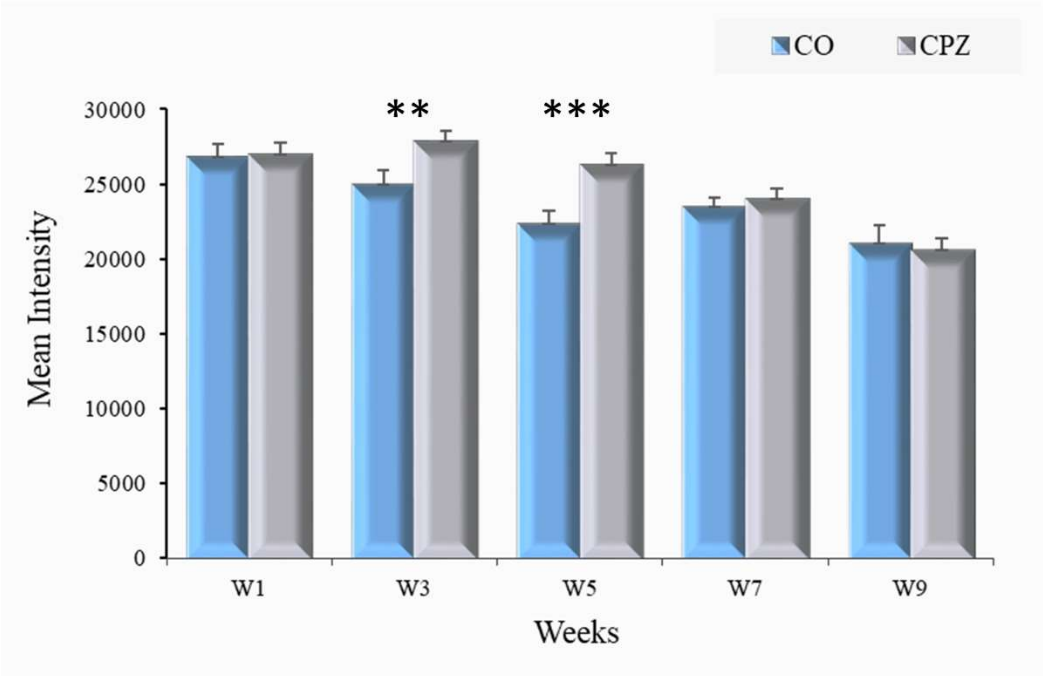

Figure S3. Formation of corpus callosum demyelination by CPZ treatment in the CO and CPZ group. LFB/CV staining for determination of myelin content by intensity measurement. Our results show that in the third and fifth weeks of the treatment, the CPZ treatment significantly reduced myelin content in the CPZ group compared to the CO group. CC: corpus callosum; CO: control group; CPZ: cuprizone group; LFB: luxol fast blue; W: week; **: $p < 0.01$ vs. CO; ***: $p < 0.001$ vs. CO. The data are presented as mean ± SEM.
